# Effects of Selenium Dietary Yeast on Growth Performance, Slaughter Performance, Antioxidant Capacity, and Selenium Deposition in Broiler Chickens

**DOI:** 10.3390/ani13243830

**Published:** 2023-12-12

**Authors:** Jinmei Liu, Zheng Wang, Chong Li, Zhimin Chen, Aijuan Zheng, Wenhuan Chang, Guohua Liu, Huiyi Cai

**Affiliations:** 1 Key Laboratory for Feed Biotechnology of the Ministry of Agriculture and Rural Affairs, Institute of Feed Research, Chinese Academy of Agriculture Sciences, Beijing 100081, China; ljm9768@163.com (J.L.); masterli1989@sina.com (C.L.); chenzhimin@caas.cn (Z.C.); zhengaijuan@caas.cn (A.Z.); caihuiyi@caas.cn (H.C.); 2 College of Life Science and Technology, Beijing University of Chemical Technology, Beijing 100029, China; wangzheng@mail.buct.edu.cn

**Keywords:** yeast selenium, broiler, growth performance, carcass quality, antioxidant capacity, selenium deposition

## Abstract

**Simple Summary:**

Se yeast is a microbial fermentation product created by adding sodium selenite in the yeast cultivation process, which is a superior product of an organic Se source. It exhibits greater bioavailability than the product from inorganic Se sources, and the increased Se levels are maintained for a longer period after the supplementation has ceased. This study evaluated the effects of Se yeast on the growth performance, slaughter performance, antioxidant capacity, and Se deposition in broilers. In conclusion, dietary supplementation of Se yeast can lead to reduced abdominal fat percentages, improved antioxidation function, and increased Se deposition in broiler tissues, thereby enhancing the meat quality of broilers, which holds great significance for the precise production of Se-enriched functional chicken products.

**Abstract:**

Selenium (Se) yeast, a bioavailable form of selenium, exhibits enhanced bioavailability due to its unique organic matrix and superior metabolic availability compared to the inorganic selenium sources. This study aims to evaluate the effects of Se yeast on the growth performance, slaughter performance, antioxidant capacity, and Se deposition in broiler chickens. A total of 264 1-day-old male AA broilers (38.7 ± 0.1 g) were randomly assigned to four treatment groups, with six replicates of 11 chickens per replicate. The broilers were fed a basal diet or a diet supplemented with 0.1, 0.2, and 0.4 mg/kg Se yeast. The experiment lasted for 42 days. Although the results showed that Se yeast did not significantly improve the growth performance of broilers, it did significantly decrease the abdominal fat ratio. Additionally, supplementation of Se yeast significantly improved the antioxidant capacity of broilers. The quadratic regression models were used to simulate the relationship between Se content in the feed and Se deposition in broiler tissues. The regression equations were as follows: pectoral muscle, Y = 2.628X − 0.340X^2^ − 0.592 (R^2^ = 0.927); leg muscle, Y = 2.317X − 0.272X^2^ − 0.490 (R^2^ = 0.937); liver, Y = 3.357X − 0.453X^2^ − 0.493 (R^2^ = 0.961); kidney, Y = 4.084X − 0.649X^2^ + 0.792 (R^2^ = 0.932). Based on these findings, the Se deposition in broiler tissues can be predicted by the Se content of the additive, which is of great significance for the precise production of Se-enriched functional chicken products.

## 1. Introduction

Selenium (Se) is considered a necessary micronutrient element for almost all life forms. In recent decades, the response of broilers to dietary selenium supplementation levels and sources has received considerable attention. In fact, the grains used in the actual feed contain Se, 50% of which is in the form of selenomethionine (Se-Met); however, as the Se content of plant grains is affected by the Se content of the soil, the poultry industry needs Se supplements to avoid nutritional deficiencies [1]. It is a crucial trace element necessary for animal growth, playing key roles in animal development, immunity, reproduction, and antioxidation [2,3]. Research indicates that Se, after being absorbed, exists in the form of selenocysteine in the body, which serves as the active center in 21 essential amino acids and 25 Se-containing proteins [4]. Selenocysteine is also a vital component of enzymes such as glutathione peroxidase, thioredoxin reductase, and thyroglobulin deiodinase. Se deficiency may cause many diseases in livestock, such as poor growth, liver necrosis, pancreatic fibrosis, leucomyopathy, exudative quality disorders, hypoimmunity, hypothyroidism, and reduced fertility and hatchability [5]. Therefore, selenium is indispensable in animal diets. The efficiency of Se depends on the level and form of dietary Se. For instance, organic Se sources are more effective as antioxidant modulators compared to sodium selenite [1].

Se yeast is a microbial fermentation product created by adding sodium selenite to the yeast cultivation process, which is a superior product of an organic Se source [6]. It was reported that Se from Se yeast exhibited greater bioavailability than Se from inorganic Se sources, and increased Se levels are maintained for a longer period after supplementation has ceased [7,8]. Additionally, Se present in the selenium yeast is an organic structure which is less toxic, easier to digest, and easier to be absorbed and used by the body [9] than inorganic selenium. Organic selenium supplements are more effective than inorganic selenium supplements at improving broiler growth performance [10], because selenium yeast is more likely to promote GSH-Px activity and selenium deposition in the muscle of broilers [11,12,13,14]. The bioefficacy of different Se additives may be assessed by the deposition of Se in different animal tissues. It has been shown in various studies that the Se deposition in the muscles could be significantly enhanced by using organic Se forms [15,16,17]. However, there were only a few systematic studies on organic Se in broiler production. The purpose of this study was to investigate the effects of different levels of Se yeast in grain feed on the growth performance, slaughter performance, antioxidant capacity, and relationship between Se content in feed and Se deposition in broiler tissue. Se yeast in the form of organic matter in grains used to feed broilers was provided by the Beijing University of Chemical Technology. This study provides a theoretical basis for the rational use of Se yeast and technical support for the production of functional Se-enriched meat.

## 2. Materials and Methods

### 2.1. Bird Management

A total of 264 1-day-old Arbor Acres mail broilers were randomly divided into four treatment groups with six replicates of 11 chickens per replicate. As shown in Table 1, the basal diets, including the starter (1–21 d) and the grower (22–42 d) phases (Table 1), were formulated according to the nutrient requirements for broilers recommended by the Ministry of Agriculture of the People’s Republic of China (2004).Broilers were fed either a basal diet or a diet supplemented with 0.1, 0.2, and 0.4 mg/kg (Table 2). Se yeast in the basic diet. The Se yeast was a fermentation product of poplar sawdust from the Beijing University of Chemical Technology, 69.4 ± 0.4 g/L dry cell weight, 2000 ± 17 g/g Se content, and 92% organic Se content. All birds were raised in three-layer cages with ad libitum access to feed and water, 16 h of light and 8 h of darkness per day, and controlled ventilation. The temperature was maintained at 35 °C for the first 3 d and then gradually reduced to a constant temperature of 25 °C according to normal management practices. The relative humidity was kept at 70% during the first week, and thereafter at about 60%. Broilers were vaccinated for Newcastle disease and infectious bronchitis disease at hatching, 7th day, and 21st day. All experimental procedures were approved by the Animal Ethics Committee of the Chinese Academy of Agricultural Sciences (AEC-CAAS-20191106) and performed according to the guidelines for animal experiments set by the National Institute of Animal Health.

### 2.2. Sampling

On the 21th and 42th day, following 8 h of fasting, all broilers were weighed, and feed intake was measured on a per-cage basis. The average daily gain (ADG), average daily feed intake (ADFI), and feed to gain (F/G) were calculated. One 42 day-old broiler with average body weight (BW) was selected from each duplicate cage and weighed, electrically stunned, and manually slaughtered within 5 min. The blood of broilers was withdrawn after stunning by cardiac puncture, placed into EDTA anticoagulated tubes, centrifuged at 1300× *g* for 10 min at 4 °C, and stored at −20 °C to determine serum biochemical indices. The weight of the carcass, eviscerated, pectoral muscle, leg muscle, and abdominal fat was recorded. The percentage by weight of the carcass, eviscerated, pectoral muscle, leg muscle, abdominal fat, liver, kidney, spleen, thymus, and bursa Fabricius to live body weight was determined. The left pectoralis and leg muscles, liver, and kidney were sampled, immediately frozen in liquid nitrogen, and stored at −20 °C for analysis of Se content in the muscle of broilers, and the right for drip loss, cooking loss, pH, and meat color determination [18].

### 2.3. Measurement of Meat Quality

The pH value was measured at 45 min and 24 h postmortem by direct insertion of an electrode (Testo 205, Testo AG, Lenzkirch, Germany) 2 cm deep into the breast muscle (measured three times at three different places around the meat sample and averaged). The water-holding capacity of meat was estimated by determining drip loss and cooking loss as described by Zhuang and Savage [19]. Meat color was measured at 45 min and 24 h after slaughter by a colorimeter (Minolta CR-400, Konica Minolta Sensing, Osaka, Japan) using the CIELAB trichromatic system as lightness, redness, and yellowness, and the aver-age color value was based on three recordings on the same muscle.

### 2.4. Antioxidant Index Analysis

Serum total antioxidant capacity (T-AOC) and activity of superoxide dismutase (SOD) and the content of malondialdehyde (MDA) were determined by commercial kits (Nanjing Jian cheng Bioengineering Institute, Nanjing, China) using the photo colorimetric method, hydroxylamine method, and TBA method, respectively.

### 2.5. Se Analysis

The Se content in the liver, kidney, pectoral muscle, and leg muscle was determined according to GB 5009.93-2010 [20] recommended by the State Food and Drug Administration and State Health and Family Planning Commission; the Se content in the feed was determined according to GB/T 13883-2008 [21] recommended by the State Standardization Management Committee of the General Administration of Quality Supervision and Inspection and Quarantine of the People’s Republic of China. All the instruments used were atomic fluorescence spectrometers (AFS-930, Beijing Jitian Instrument Co., Ltd., Beijing, China).

### 2.6. Statistical Analysis

The data were analyzed by using General Linear Model procedure of the SPSS16.0 software package for Windows (SPSS Inc, Chicago, IL, USA). Significant differences between treatment means were separated using the Duncan’s multiple range test. The results are presented as the mean and standard error of the mean (SEM). All statements of significance are based on a probability of *p* < 0.05.

## 3. Results

### 3.1. Growth Performance

The effects of yeast Se supplementation levels on growth performance is shown in Table 3. The supplementation of yeast Se did not have a significant effect on the ADG, ADFI, F/G, or BW of broilers (*p* > 0.05).

### 3.2. Slaughter Performance

There were no significant differences observed in most of the examined parameters of carcass characteristics between the control and experimental groups in Table 4 (*p* > 0.05). However, the groups supplementing with yeast Se demonstrated significantly lower abdominal fat percentages compared to the control group (*p* < 0.05).

### 3.3. Meat Quality

As presented in Table 5, there were no significant differences in drip loss, cooking loss, pH, or the color of meat at 45 min and 24 h after slaughter between the control and experimental groups (*p* > 0.05)

### 3.4. Serum Antioxidant Indexes

The serum GSH levels of broilers were significantly improved by the supplementation of yeast Se (*p* = 0.05, Table 6). There was a tendency of increasing levels of serum T-AOC, SOD, CAT, and decreasing MDA levels by rising the Se level in the diets (*p* > 0.05).

### 3.5. Se Deposition in Tissues

As shown in Table 7, the dietary supplementation of yeast Se at different levels significantly increased Se contents in the breast muscle, leg muscle, liver, and kidney of broilers (*p* < 0.05). The deposition pattern of Se in broiler tissues was as follows: kidney > liver > pectoral muscle > leg muscle for early stage (1–21 days of age), kidney > liver > leg muscle > pectoral muscle for later stages (22–42 days of age).

### 3.6. Regression Equation between Se Levels in Feed and Tissue

The regression equations between Se levels in feeds and tissues showed in Table 8 and Table 9. The Se content of broiler tissue presented significant linear and secondary dose response (*p* < 0.05), of those, the simulation effect of quadratic regression equation was better (*p* < 0.05).

## 4. Discussion

Se plays a vital role in animal growth as it is involved in the enzyme group of iodothyroninedeiodinases, which are responsible for the metabolism of thyroid hormones necessary for normal growth and development [22]. In the present study, no significant differences were found in ADG, BW, ADFI, and F/G of broiler chickens between the control and test groups. However, broilers’ BW and ADFI tended to go up with larger doses of yeast Se treatment. These results are in line with what other studies have reported [23,24,25]. However, Marković et al. found that supplementation of Se improved BW, WG, and FCR in the broilers [26]. This improvement could be attributed to the enhanced activation of thyroid hormones due to increased Se content [27]. Therefore, increasing the Se supplementation levels in our experiments may yield similar results.

Our study yielded no significant differences in the majority of the carcass features that were investigated between the control and experimental groups, which aligns with the findings reported by Payne and Southern [23] that carcass traits were not affected by the Se source or level of supplementation. In contrast, Upton et al. [28] showed that Se-enriched yeast supplementation (0 to 0.2 mg/kg) boosted various characteristics of cutup yields of high-yielding broilers. The abdominal fat weight were significantly lower in broiler chickens fed the diet containing nano-Se than those chickens fed the control diet [29]. In our study, compared to the control group, the abdominal fat ratio (AFR) of the treatment groups was significantly decreased, and the 0.1 mg/kg treatment group reduced it effectively. The lower the AFR, the better the carcass quality. It is suggested that an appropriate amount of Se supplementation should be added to the feed, which can promote the digestion and absorption of fat in the intestinal tract and reduce the accumulation of fat in the body, effectively improve slaughtering performance.

Water-holding capacity, pH value, and color are the significant assessment indicators of meat quality. After the animals were slaughtered, the blood circulation in the muscle stopped, resulting in a large accumulation of lactic acid, thus reducing the value of the pH. The delayed decrease in pH value will lead to the decrease in protein denaturation, thus improving the water-retention capacity of the muscle [30]. The experiment’s findings showed that there were no notable variations between the control and experimental groups in terms of drip loss, cooking loss, pH, or meat color at 45 and 24 h following slaughter. However, the drip loss tended to decrease, and the effect of the 0.4 mg/kg addition group was the best. Payne and Southern [18] also proved that the 24 h breast moisture loss of broilers was not affected by Se supplementation sources of yeast Se. However, broilers ingesting organic Se in their diets showed a lower drip loss, according to Wang et al. [31]. And it was also reported that organic selenium might improve meat quality by reducing the drip loss from poultry meat [32]. The disagreement in results may be due to the differences in animal species, Se sources, and addition level. According to several reports, adding nano-Se could lessen the loss of chicken muscle drip [32,33]. The ability of muscle proteins to attract water and hold it within the cells is of great importance to meat quality. Selenium is essential to the body’s intracellular and extracellular antioxidant systems [34,35]; the improved antioxidant status may promote the maintenance of cell membrane integrity [36]. M.J.A. et al. also found that a lower pH also decreases the muscle protein ability to bind to water, causing shrinkage of the myofibrils [37], which could ultimately result in reduced drip loss [38].

Antioxidant capacity is an important determinant of animal health and the quality of animal products. The antioxidant capacity of chickens is regulated by the antioxidant system and a multitude of important enzymes, including T-AOC, GSH-Px, SOD, CAT, and MDA [39,40]. GSH-Px is a Se-dependent enzyme that catalyzes the reduction of H_2_O_2_ and organic peroxides to H_2_O and the corresponding stable alcohol, thus inhibiting the formation of free radicals [41]. SOD is a crucial antioxidant enzyme in organisms that helps superoxide anion dismutate into H_2_O_2_ and O_2_ [42]. MDA is one of the metabolic products of lipid peroxides, and it is negatively correlated with the GSH-Px activity [11]. Together with other compounds like glutathione peroxidases, CAT can split H_2_O_2_ into safe H_2_O and O_2_, blocking that pathway and protecting the organism [43]. The addition of selenium yeast reduces the production of oxidized products and thus improves the antioxidant capacity of broiler muscle. Our findings showed that adding yeast Se to the diet greatly raised the GSH levels in broilers. By adding different levels Se to diets, there was a tendency for T-AOC, SOD, and CAT levels to rise while MDA levels fell. And the 0.2 mg/kg treatment group had the best effect, which is consistent with the conclusion above. Our findings supported other studies that found birds fed yeast enhanced with selenium had higher antioxidant capacities than birds fed alternative sources of selenium [44].

In Se-enriched yeast, Se mainly exists as selenomethionine (SeM) and is incorporated non-specifically into peptide chains. Furthermore, it exhibits a high digestibility [45].

It is generally accepted that organic selenium has a better bioavailability and tissue retention than inorganic selenium. Therefore, selenium accumulation in tissues is a very important criterion for the use of the mineral. Testing of the selenium in chicken muscle confirmed that organic selenium (selenium yeast) has a higher absorption rate [46,47].

In the present study, the dietary supplementation of yeast Se at varying levels resulted in a significant increase in selenium contents in the breast muscle, leg muscle, liver, and kidney of broiler chickens. These findings align with several previous reports on the subject [23,48]. Payne and Southern [23] proved that broilers fed yeast Se had increased breast Se concentrations. Konkol et al. [48] tested the content of selenium in the muscles of chickens and confirmed that selenium in the organic form (selenium yeast) is better absorbed. Se and S (sulfur) have extremely similar atomic characteristics, which suggests that SeM might be integrated into proteins at a rate similar to Methionine (Met). The increase in breast Se in broilers fed Se-enriched yeast (SY) could be explained by the ability of Se to be regarded as S and the ability of SeM to replace Met so that it can be integrated into protein when digested [23]. Gul et al. [44] showed that compared to other sources of Se, birds fed Se-enriched yeast had more Se deposition. Deng et al. [49] and Bauché et al. [39] reported that dietary organic Se increased Se concentrations in the liver, breast muscles, thigh muscle, and kidney of broilers. Our study also found that the Se deposition efficiency was different between the early stage and late stage. The deposition rate of Se in broiler tissues was kidney > liver > pectoral muscle > leg muscle for the early stage (1–21 days of age) and kidney > liver > leg muscle > pectoral muscle for the late stage (22–42 days of age).

In our study, the Se content of broiler tissue presented a significant linear and secondary dose effect; of those, a quadratic regression equation simulated the results better. Such outcome supports previous research finding that Se contents in breast and thigh meat increased significantly (*p* < 0.01) with the level of Se supplementation [26]. The study found that the mRNA levels of the methionine (Met) metabolism gene glycine N-methyltranserfase (GNMT) were markedly upregulated (*p* < 0.05) in the Se-enriched yeast group, which may reveal that Se from yeast Se is deposited more efficiently than Se from sodium selenite or nano-selenium, probably via enhancing the route of Met metabolism [50].

## 5. Conclusions

This experiment evaluated the effects of yeast Se on the growth performance, slaughter performance, antioxidant capacity, and Se deposition in broilers. The results showed that dietary supplementation of yeast Se can lead to reduced abdominal fat percentages, improved antioxidation function, and increased Se deposition in broiler tissues, thereby enhancing the meat quality of broilers. Meat quality is enhanced with increasing levels of selenium addition within a certain range. The Se content of broiler tissues and the dietary Se level exhibited significant linear and quadratic dose responses, with the quadratic regression equation providing a better simulation. In summary, using the regression equation, the amount of Se deposition in broiler tissues can be calculated based on the Se content in the diet, which holds great significance for the precise production of Se-enriched functional chicken products.

## Figures and Tables

**Table 1:** Ingredients and chemical composition of basal diets for broilers.

Ingredients, %	1–21 d	22–42 d
Corn	54.81	60.43
46% Soybean meal	33.24	32.02
Soybean oil	3.98	4.05
Cottonseed meal	3.00	5.00
Dicalcium phosphate	1.88	1.75
Limestone	1.23	1.08
Premix ^1^	1.00	1.00
Salt	0.30	0.30
*L*-Lysine	0.29	0.20
*DL*-Methionine	0.27	0.17
Total	100.00	100.00
**Chemical composition**		
ME ^2^, MJ/kg	12.55	12.97
CP ^3^, %	21.68	19.37
Ca ^3^, %	0.97	0.95
Total P ^3^, %	0.84	0.78
Nonphytate phosphorus, %	0.48	0.45
Lysine ^3^, %	1.06	0.98
Methionine ^3^, %	0.48	0.40
Threonine ^3^, %	0.86	0.73
Tryptophan ^3^, %	0.24	0.22

^1^ Provided the following per kg of diet: vitamin A, 12,000 IU; cholecalciferol, 2000 IU; vitamin E (DL-α-tocopheryl acetate), 20 IU; vitamin K_3_, 2.15 mg; riboflavin, 8.00 mg; pyridoxine, 4.5 mg; vitamin B_12_, 0.02 mg; calcium pantothenate, 26 mg; nicotinic acid, 68 mg; folic acid, 1 mg; biotin, 0.20 mg; Fe, 110 mg; Cu, 8 mg; Zn, 78 mg; Mn, 105 mg; I, 0.34 mg; Se, 0.15 mg; choline chloride, 1500 mg. ^2^ Calculated value. ^3^ Analyzed value.

**Table 2:** Analyzed amount of Se fed to each group.

Items	0.0 mg/kg	0.1 mg/kg	0.2 mg/kg	0.4 mg/kg
Analyzed amount of Se (mg/kg)	0.15	0.26	0.36	0.54

**Table 3:** Effects of dietary Se supplementation levels on the growth performance of broilers.

Items		0	0.1 mg/kg	0.2 mg/kg	0.4 mg/kg	SEM	*p* Value
1–21 d	ADG, g	38.78	39.31	39.21	39.87	2.591	0.723
	ADFI, g	50.83	52.49	52.90	51.65	3.339	0.428
	F/G	1.31	1.34	1.35	1.30	0.058	0.318
22–42 d	ADG, g	90.62	91.17	95.42	96.89	6.872	0.235
	ADFI, g	146.34	146.17	152.10	157.32	11.182	0.156
	F/G	1.61	1.61	1.60	1.63	0.098	0.822
1–42 d	ADG, g	64.69	65.24	67.31	68.38	5.872	0.228
	ADFI, g	98.58	99.33	102.51	104.48	5.562	0.205
	F/G	1.53	1.52	1.53	1.53	0.057	0.998

*n* = 6. BW, body weight; ADG, average daily gain; ADFI, average daily feed intake; F/G, feed to gain.

**Table 4:** Effects of dietary Se supplementation levels on the carcass characteristics of broilers, %.

Items	0	0.1 mg/kg	0.2 mg/kg	0.4 mg/kg	SEM	*p* Value
Dressing percentage	88.26	89.59	88.68	90.56	4.984	0.406
Percentage of eviscerated yield	72.72	74.39	72.74	75.05	4.165	0.353
Percentage of breast muscle	32.20	32.55	32.63	32.23	1.628	0.981
Percentage of thigh muscle	22.31	22.06	22.00	22.11	1.235	0.994
Percentage of abdominal fat	2.15 ^a^	1.49 ^b^	1.57 ^b^	1.54 ^b^	0.113	0.021

*n* = 6. ^a,b^ means without common letters differ at *p* < 0.05.

**Table 5:** Effects of dietary Se supplementation levels on the meat quality of broilers.

Items	0	0.1 mg/kg	0.2 mg/kg	0.4 mg/kg	SEM	*p* Value
Drip loss (%)	5.47	4.81	4.88	4.64	0.283	0.886
Cooking loss (%)	21.76	20.65	20.47	20.15	1.069	0.960
pH_45min_	5.64	5.71	5.72	5.81	0.257	0.501
L_45min_	50.69	49.58	49.59	54.23	2.429	0.068
a_45min_	6.81	7.84	7.15	7.43	0.375	0.870
b_45min_	7.90	7.88	8.69	7.40	0.411	0.827
pH_24h_	5.43	5.49	5.39	5.28	0.264	0.135
L_24h_	52.75	50.46	51.26	55.96	2.509	0.101
a_24h_	3.99	4.21	4.08	3.09	0.205	0.594
b_24h_	9.06	8.33	9.00	9.93	0.428	0.652

*n* = 6. pH_45min_—pH of meat measured 45 min postmortem; pH_24h_—pH of meat measured 24 h after chilling the carcasses to 4 °C; SEM: the pooled standard error of the mean; L—lightness color parameter; a—redness color parameter; b—yellowness color parameter.

**Table 6:** Effects of dietary Se supplementation levels on the serum antioxidant capacity of broilers.

Items	0	0.1 mg/kg	0.2 mg/kg	0.4 mg/kg	SEM	*p* Value
T-AOC, U/mL	5.0	5.47	5.49	5.47	0.267	0.844
SOD, U/mL	186.86	188.66	189.58	188.32	9.382	0.760
CAT, U/L	4.63	5.24	5.49	5.24	0.273	0.657
MDA, nmoL/mL	3.31	2.92	3.11	3.23	0.156	0.076
GSH, μg/mL	574.37 ^a^	732.52 ^b^	746.22 ^b^	730.58 ^b^	36.142	0.050

*n* = 6. ^a,b^ means without common letters differ at *p* < 0.05; T-AOC, total antioxidant capacity; SOD, superoxide dismutase; CAT, catalase; MDA, malondialdehyde; GSH, glutathione.

**Table 7:** Effects of dietary Se supplementation levels on the Se levels of broiler tissues (%).

Items	0	0.1 mg/kg	0.2 mg/kg	0.4 mg/kg	SEM	*p* Value
Breast muscle	0.39 ^a^	1.56 ^b^	3.68 ^c^	4.44 ^d^	0.121	<0.001
Thigh muscle	0.43 ^a^	1.32 ^b^	3.49 ^c^	4.37 ^d^	0.118	<0.001
Liver	1.56 ^a^	3.74 ^b^	5.50 ^c^	6.67 ^d^	0.873	<0.001
Kidney	2.11 ^a^	4.53 ^b^	6.54 ^c^	6.76 ^d^	0.259	<0.001

*n* = 6. ^a,b,c,d^ means without common letters differ at *p* < 0.05.

**Table 8:** Regression equation between the Se level in feed and tissues of 21-day-old broilers.

Items	Model	Equation	R2	*p* Value
Breast muscle	Linear regression	Y = 1.116X + 0.426	0.838	<0.001
	Quadratic regression	Y = 2.628X − 0.340X^2^ − 0.592	0.927	<0.001
Thigh muscle	Linear regression	Y = 1.107X + 0.325	0.876	<0.001
	Quadratic regression	Y = 2.317X − 0.272X^2^ − 0.490	0.937	<0.001
Liver	Linear regression	Y = 1.313X + 1.940	0.845	<0.001
	Quadratic regression	Y = 3.357X − 0.453X^2^ − 0.493	0.961	<0.001
Kidney	Linear regression	Y = 1.156X + 2.866	0.685	<0.001
	Quadratic regression	Y = 4.084X − 0.649X^2^ + 0.792	0.932	<0.001

**Table 9:** Regression equation between the Se level in feed and tissues of 42-day-old broilers.

Items	Model	Equation	R2	*p* Value
Breast muscle	Linear regression	Y = 1.070X + 0.181	0.936	<0.001
	Quadratic regression	Y = 1.547X − 0.189X^2^ + 0.013	0.946	<0.001
Thigh muscle	Linear regression	Y = 1.066X + 0.346	0.944	<0.001
	Quadratic regression	Y = 1.183X − 0.048X^2^ + 0.308	0.945	<0.001
Liver	Linear regression	Y = 2.340X + 0.815	0.867	<0.001
	Quadratic regression	Y = 2.450X − 0.040X^2^ + 0.776	0.867	<0.001
Kidney	Linear regression	Y = 2.394X + 1.436	0.945	<0.001
	Quadratic regression	Y = 2.311X − 0.031X^2^ + 1.468	0.945	<0.001

## Data Availability

The datasets used and analyzed during the current study are available from the corresponding author on reasonable request.
